# Maxillary avascular necrosis after Le Fort I osteotomy: a systematic review of ischemic complications

**DOI:** 10.1186/s40902-026-00523-x

**Published:** 2026-08-19

**Authors:** Chung-woo Lee, Sung-Yun Hwang, Ladan Esmaeili, Dae-Seok Hwang

**Affiliations:** 1 Department of Oral and Maxillofacial Surgery, Dental and Life Science Institute, School of Dentistry,, Pusan National University, Yangsan, Korea, Republic of; 2 Department of Oral and Maxillofacial Surgery, Pusan National University Dental Hospital, Yangsan, Korea, Republic of

**Keywords:** Le Fort I osteotomy, Maxilla, Avascular necrosis, Orthognathic surgery, Descending palatine artery, Ischemic complication, Collateral circulation, Systematic review

## Abstract

**Background:**

Maxillary avascular necrosis (AVN) after Le Fort I osteotomy is rare but potentially devastating. Although injury to the descending palatine artery has traditionally been regarded as a major cause, the relative contribution of individual vascular and surgical factors remains unclear. This systematic review aimed to synthesize the clinical evidence on maxillary AVN after orthognathic surgery and to evaluate whether postoperative ischemic complications are better explained by isolated arterial injury or cumulative vascular compromise.

**Main body:**

A systematic literature search of PubMed, Scopus, Web of Science, and Embase was performed from database inception to May 2026 in accordance with PRISMA 2020 guidelines. English-language retrospective cohort studies, prospective cohort studies, and case series reporting maxillary AVN or ischemic complications associated with orthognathic surgery were included. Single-case reports, reviews, conference abstracts, editorials, and studies with insufficient clinical data were excluded. Seven studies were included in the qualitative synthesis. Reported ischemic manifestations ranged from tooth discoloration and pulpal necrosis to gingival necrosis, dentoalveolar sequestration, partial maxillary necrosis, and anterior segment AVN. Among cohort studies, clinically significant ischemic complications were uncommon, with reported rates ranging from 0.2% to 3.3%, whereas tooth discoloration reflecting pulpal ischemia was reported in 3.4% of orthognathic surgery patients. Segmented or multisegment Le Fort I osteotomy, transverse expansion, large maxillary advancement, palatal soft-tissue compromise, cleft-related scarring, and previous palatal surgery were repeatedly described among affected patients. However, no single factor consistently explained all reported cases. The relationship between descending palatine artery injury and ischemic complications was inconsistent, and ligation alone was not uniformly associated with AVN.

**Conclusion:**

Maxillary AVN after Le Fort I osteotomy is an exceptionally rare but serious complication. The available evidence supports a multifactorial mechanism in which multiple vascular insults cumulatively impair collateral perfusion rather than a mechanism driven by isolated descending palatine artery injury. Surgical prevention should therefore emphasize preservation of overall maxillary perfusion and collateral soft-tissue attachments.

**Supplementary Information:**

The online version contains supplementary material available at 10.1186/s40902-026-00523-x.

## Introduction

Le Fort I osteotomy has become the standard procedure for correction of maxillary dentofacial deformities because it permits accurate three-dimensional repositioning of the maxilla while providing stable functional and esthetic outcomes [1, 2]. Continuous refinements in surgical planning and fixation techniques have resulted in a highly predictable procedure with a relatively low incidence of major complications [3, 4]. 

Despite its overall safety, ischemic injury to the mobilized maxilla remains one of the most devastating complications following Le Fort I osteotomy because it may result in extensive bone and soft-tissue necrosis, prolonged treatment, secondary reconstruction, and permanent functional impairment [5]. 

Maintenance of maxillary viability after osteotomy depends on preservation of an adequate vascular supply during mobilization and subsequent healing [6]. Unlike many skeletal segments, the Le Fort I segment is perfused through a rich collateral vascular network rather than a single dominant arterial pedicle [7]. 

Cadaveric and anatomical studies have demonstrated that perfusion of the mobilized maxilla is maintained through multiple vascular sources, including the descending palatine artery (DPA), ascending palatine artery, ascending pharyngeal artery, posterior superior alveolar vessels, and the surrounding palatal and buccal soft tissues [7, 8]. These anatomical findings suggest considerable redundancy within the maxillary circulation and provide a biological explanation for the rarity of clinically significant ischemic complications [6]. 

Historically, the DPA has been regarded as the principal vascular pedicle of the Le Fort I segment, leading many surgeons to advocate meticulous preservation of the vessel whenever possible [6]. However, experimental evidence has questioned whether injury to this vessel alone is sufficient to compromise maxillary viability [9]. In a primate model, Bell and colleagues demonstrated that interruption of the descending palatine vessels did not result in maxillary osteonecrosis when the surrounding soft-tissue pedicles remained intact [9]. Likewise, prospective intraoperative perfusion measurements showed that ligation of the DPA did not significantly reduce gingival blood flow after Le Fort I osteotomy, further supporting the importance of collateral circulation [10]. 

Recent anatomical investigations have also demonstrated substantial interindividual variation in the vascular anatomy of the Le Fort I segment, indicating that collateral perfusion may differ considerably among patients [11]. This variability raises important questions regarding how individual vascular injuries interact with surgical and patient-related factors in the development of postoperative ischemic complications [11]. 

Clinically, postoperative ischemic complications represent a spectrum of tissue hypoperfusion ranging from transient pulpal ischemia and tooth discoloration to gingival necrosis, dentoalveolar sequestration, partial maxillary necrosis, and complete avascular necrosis of the mobilized segment [12]. Although these complications are uncommon, they are associated with considerable morbidity and frequently require prolonged wound care, endodontic treatment, sequestrectomy, bone grafting, or secondary reconstructive procedures [13]. 

Several mechanisms have been proposed to explain postoperative maxillary avascular necrosis, including excessive maxillary advancement, multisegment osteotomy, transverse expansion, palatal soft-tissue injury, vascular thrombosis, previous palatal surgery, and disruption of collateral circulation [12]. However, the relative contribution of these factors remains poorly defined because the available evidence has largely been derived from isolated case reports and small observational studies [13]. 

Previous systematic reviews have summarized avascular necrosis or osteonecrosis after orthognathic surgery, including reported cases together with observational studies [12, 13]. However, the available evidence specifically derived from cohort studies and case series remains limited, and the relative contribution of individual surgical and vascular factors to maxillary ischemic complications after Le Fort I osteotomy remains unclear [12, 13]. Therefore, this systematic review aimed to synthesize cohort studies and case series reporting maxillary avascular necrosis or ischemic complications after orthognathic surgery, with particular emphasis on clinical manifestations, incidence, proposed risk factors, management strategies, outcomes, and the role of descending palatine artery injury versus cumulative vascular compromise.

## Materials and methods

### Literature search

A systematic literature review was conducted in accordance with the Preferred Reporting Items for Systematic Reviews and Meta-Analyses (PRISMA) 2020 guidelines [14]. Four electronic databases (PubMed, Scopus, Web of Science, and Embase) were searched from database inception to May 2026 to identify studies reporting avascular necrosis of the maxilla associated with orthognathic surgery. Only articles published in English were included.

The search strategy combined controlled vocabulary (Medical Subject Headings [MeSH] in PubMed) and free-text terms using the Boolean operators OR and AND. MeSH terms and corresponding free-text keywords related to maxilla, orthognathic surgery, and avascular necrosis were identified. Synonymous terms within each concept were combined using OR, and the three concept groups were subsequently combined using AND. Equivalent search strategies were adapted for Scopus, Web of Science, and Embase using the appropriate database-specific syntax. The search strategy was as follows: [(maxilla OR maxillary OR jaw OR palate OR palatal) AND (orthognathic surgery OR Le Fort I OR LeFort I OR Le Fort 1 OR LeFort 1 OR maxillary osteotomy OR anterior maxillary osteotomy) AND (avascular necrosis OR aseptic necrosis OR ischemic necrosis OR ischaemic necrosis OR ischemia OR ischaemia OR ischemic OR ischaemic OR necrosis OR vascular complication OR vascular complications)].

### Eligibility criteria

Studies were eligible for inclusion if they (1) reported avascular necrosis of the maxilla associated with orthognathic surgery, (2) provided sufficient clinical information regarding patient characteristics, surgical procedures, management, or outcomes, and (3) were retrospective cohort studies or case series.

Studies were excluded if they (1) were not relevant to the review question, (2) contained insufficient data for analysis, (3) were single-case reports, (4) were review articles, editorials, conference abstracts, or letters, or (5) were not published in English. Single-case reports were excluded to improve the methodological consistency of the included evidence and to minimize the influence of anecdotal findings.

### Study selection and data extraction

After duplicate records were removed, two independent reviewers screened the titles and abstracts of all retrieved studies according to the predefined eligibility criteria. Potentially eligible articles subsequently underwent independent full-text assessment by the same reviewers.

Data were extracted using a standardized data extraction form, including the first author, publication year, country, study design, sample size, patient demographics, type of orthognathic surgery, clinical presentation, onset of avascular necrosis, suspected risk factors, treatment modalities, and clinical outcomes.

**Fig. 1:**
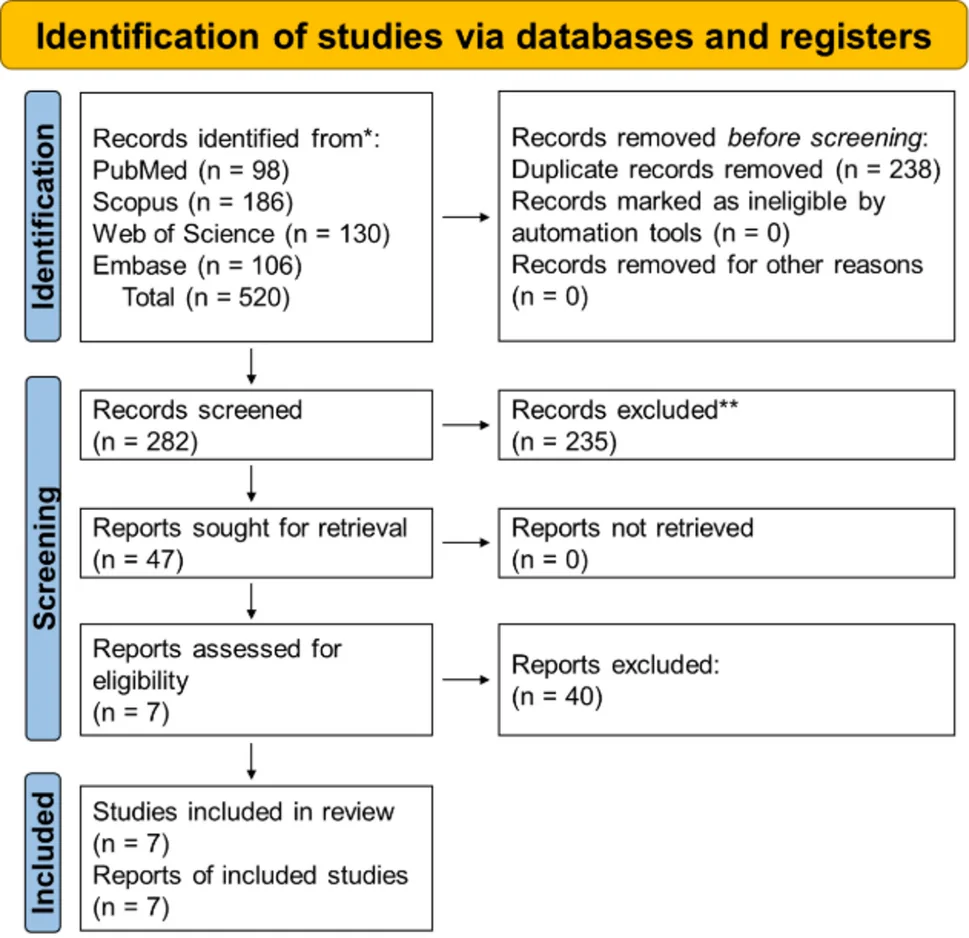
PRISMA 2020 flow diagram

Any disagreements regarding study selection or data extraction were resolved through discussion. Any unresolved disagreements were adjudicated by the corresponding author. The study selection process is summarized in the PRISMA 2020 flow diagram (Fig. 1).

### Risk of bias assessment

The methodological quality of the included studies was independently assessed by three reviewers using the Joanna Briggs Institute (JBI) Critical Appraisal Checklist for Case Series and the JBI Critical Appraisal Checklist for Cohort Studies, as appropriate [15, 16]. Disagreements were resolved through discussion, and any unresolved disagreements were adjudicated by the corresponding author. The complete appraisal checklists and study-level assessments are presented in Supplementary Tables S1 and S2.

### Data synthesis

Because of substantial heterogeneity in study design, patient populations, definitions of ischemic complications, and outcome reporting among the included studies, a quantitative meta-analysis was not considered appropriate. Therefore, the findings were synthesized qualitatively using a descriptive approach, with emphasis on study characteristics, reported ischemic complications, surgical risk factors, management of the descending palatine artery, treatment strategies, and clinical outcomes.

## Results

### Study selection

The literature search identified 520 records from four electronic databases (PubMed, Scopus, Web of Science, and Embase). After removal of 238 duplicate records, 282 records underwent title and abstract screening. Of these, 235 records were excluded. Full texts were sought and retrieved for 47 potentially eligible articles. After full-text assessment, 40 articles were excluded because they were not relevant to the review question, contained insufficient data, or were single-case reports. Finally, seven studies were included in the qualitative synthesis.

### Study characteristics

The characteristics of the included studies are summarized in Table 1. The included literature consisted of one questionnaire-based case series, five retrospective cohort studies, and one prospective cohort study [17–23]. Sample sizes ranged from 36 reported cases of established maxillary aseptic necrosis to 1455 patients undergoing orthognathic surgery [17, 20]. Two studies focused specifically on cleft populations [21, 22]. One study evaluated tooth discoloration and pulpal necrosis after orthognathic surgery [20]. The remaining studies reported intraoperative or postoperative complications following Le Fort I osteotomy [17–19, 23]. 

**Table 1:** Characteristics of the included studies

Study	Design	Population	Sample size	Primary outcome
Lanigan et al. (1990) [17]	Questionnaire-based case series	Reported AVN after maxillary osteotomy	36 AVN cases	Established maxillary AVN
de Mol van Otterloo et al. (1991) [18]	Retrospective cohort	Le Fort I osteotomy	410	Postoperative ischemic complications
Kramer et al. (2004) [19]	Prospective cohort	Le Fort I osteotomy	1000	Ischemic complications
Lee et al. (2016) [20]	Retrospective cohort	Orthognathic surgery	1455	Tooth discoloration / pulpal ischemia
Moran et al. (2018) [21]	Retrospective cohort	Cleft Le Fort I	79	Postoperative complications
Heggie et al. (2021) [22]	Retrospective cohort	Cleft maxillary advancement	207	Vascular compromise
Tai et al. (2022) [23]	Retrospective cohort	Segmented Le Fort I	120	Anterior segment AVN

*AVN* avascular necrosis

### Spectrum of ischemic complications

The reported ischemic manifestations varied from mild dental findings to partial loss of maxillary hard and soft tissues (Tables 2) [17–23]. Dental manifestations included tooth discoloration, loss of tooth vitality, pulpal necrosis requiring endodontic treatment, and tooth loss in severe cases [17, 18, 20–23]. Soft-tissue manifestations included gingival ischemia, gingival recession, soft-tissue necrosis, and exposed bone [17, 19, 21–23]. More severe osseous manifestations included buccal cortical bone necrosis, dentoalveolar sequestration, partial maxillary or alveolar necrosis, anterior segment avascular necrosis, and loss of dentoalveolar segments [17–19, 21–23]. 

**Table 2:** Clinical manifestations and outcomes of ischemic complications

Study	Ischemic events	Dental findings	Soft-tissue findings	Osseous findings	Treatment	Outcome
Lanigan [17]	36 (100%)*	Tooth devitalization; tooth loss	Gingival necrosis	Maxillary/dentoalveolar necrosis	Conservative to resection	Variable healing
de Mol [18]	1 (0.2%)	Canine devitalization	NR	Buccal cortical necrosis	Conservative	Healed
Kramer [19]	10 (1.0%)	NR	Gingival retraction	2 partial maxillary/alveolar necrosis	Conservative ± debridement	Generally favorable
Lee [20]	49 (3.4%)†	Tooth discoloration; pulpal necrosis	None	None	Root canal treatment	Tooth preservation
Moran [21]	2 (2.5%)	Loss of vitality	Localized gingival necrosis	Partial anterior AVN	Debridement; grafting	Recovered
Heggie [22]	5 (2.4%)	Tooth loss (3/5)	Gingival/mucosal loss	Maxillary bone necrosis	Debridement/reconstruction	Stable healing
Tai [23]	4 (3.3%)	Tooth loss in severe cases	Periodontal necrosis	Anterior segment AVN	Conservative or sequestrectomy	Variable

*AVN* avascular necrosis, *NR *not reported

*Lanigan included only established AVN cases

†Lee evaluated pulpal ischemia rather than clinically evident maxillary AVN

### Incidence within cohort studies

Among cohort studies that reported a denominator, ischemic complications were uncommon but clinically important. de Mol van Otterloo et al. reported one necrosis case among 410 Le Fort I osteotomies (0.2%) [18]. Kramer et al. reported ischemic complications in 10 of 1000 patients (1.0%), including eight gingival retractions and two cases of partial maxillary/alveolar necrosis [19]. In cleft cohorts, Moran et al. reported two ischemic-type events among 79 patients (2.5%) [21]. Heggie et al. identified five cases of vascular compromise among 207 cleft patients (2.4%) [22]. Tai et al. reported avascular necrosis of the maxillary anterior osteotomy segment in four of 120 patients (3.3%) [23]. Lee et al. reported tooth discoloration in 49 of 1455 orthognathic surgery patients (3.4%) [20]. However, this outcome reflected pulpal ischemia rather than clinically evident maxillary bone or soft-tissue avascular necrosis [20]. 

### Surgical factors

Segmented or multisegment Le Fort I osteotomy was the most consistently reported surgical characteristic among patients with ischemic complications (Table 3) [17, 19, 22, 23]. Additional factors repeatedly described across the included studies included transverse expansion, large maxillary advancement, and palatal soft-tissue compromise [17, 19, 21, 22]. Kramer et al. reported that all patients with ischemic complications had one or more recognized surgical or anatomical risk factors, including anterior advancement greater than 9 mm, transverse segmentation, or major anatomical irregularities [19]. In contrast, Lee et al. found no significant association between maxillary anterior multisegmental osteotomy and maxillary tooth discoloration, suggesting that pulpal ischemia and clinically evident maxillary AVN should be interpreted as related but distinct outcomes [20]. 

**Table 3:** Evidence regarding the role of the descending palatine artery (DPA) in maxillary ischemic complications

Study	Clinical evidence	Authors’ findings	Contribution to current review
Lanigan [17]	Mixed observations	DPA may contribute with other vascular insults	Supports multifactorial mechanism
de Mol [18]	Six DPA ligations without AVN	Ligation alone did not impair maxillary viability	Strong evidence against isolated DPA mechanism
Kramer [19]	AVN despite no recognized palatal vessel injury	Other vascular factors implicated	Supports cumulative vascular-insult model
Lee [20]	DPA ligation associated with tooth discoloration	Pulpal ischemia, not maxillary AVN	Dental ischemia should be interpreted separately
Moran [21]	No direct DPA evaluation	Preservation recommended	Insufficient direct evidence
Heggie [22]	No direct DPA evaluation	Previous palatal surgery emphasized	Collateral circulation likely important
Tai [23]	DPA transection documented	No independent association with AVN identified	DPA preservation recommended, causality unproven

*AVN* avascular necrosis, *DPA *descending palatine artery

### DPA and palatal vascular factors

The relationship between DPA sacrifice and ischemic complications was inconsistent across studies (Table 4) [18–20, 23]. Lee et al. found that DPA ligation was significantly associated with maxillary tooth discoloration [20]. However, de Mol van Otterloo et al. described six cases of greater palatine artery bleeding controlled by ligation without ischemic sequelae [18]. Kramer et al. reported ischemic complications in the absence of recognized intraoperative palatal vessel damage [19]. Tai et al. reported DPA transection in six patients in the overall cohort, but individual overlap with AVN was not provided [23]. Collectively, the available evidence suggests that DPA injury alone does not consistently explain postoperative maxillary AVN and is more likely to contribute in combination with other vascular risk factors [18–20, 23]. 

**Table 4:** Cross-study evidence synthesis of reported surgical and vascular risk factors

Risk factor	Lanigan [17]	de Mol [18]	Kramer [19]	Lee [20]	Moran [21]	Heggie [22]	Tai [23]
Segmented/multisegment Le Fort I	Associated	Present	Associated	No association	Not evaluated	Described	Associated
Transverse expansion	Associated	NR	Associated	NR	NR	Described	Procedure-specific
Superior repositioning/impaction*	Associated	Described	Described	No association	NR	Described	NR
Large advancement	Described	NR	Associated (> 9 mm)	No association	Described (9–10 mm)	NR	Not identified
DPA injury/ligation	Mixed findings	Six ligations without ischemia	No recognized vessel injury	Associated with tooth discoloration	Not evaluated	Not evaluated	Reported; no association with AVN shown
Palatal injury/scarring	Associated	Suggested	Suggested	NR	Associated	Associated	Palatal perforation linked to DPA injury
Previous palatal surgery/cleft	Reported	No	No	Excluded	Associated	Associated (all cases)	Excluded

*AVN* avascular necrosis, *DPA *descending palatine artery, *NR *not reported

*Superior repositioning/impaction was inconsistently reported and was not interpreted as an independent risk factor. Lee et al. evaluated pulpal ischemia rather than maxillary AVN

### Cleft-related findings

The two cleft-specific studies consistently identified cleft-related palatal scarring and previous palatal surgery, including palatal transposition flap or pharyngeal flap procedures, as important contextual factors [21, 22]. In Heggie et al., all five affected patients had undergone previous palatal surgery or pharyngeal flap surgery before maxillary advancement, and two underwent segmental expansion [22]. Three of the five eventually lost teeth, whereas two retained their dentition with stable mucosal or gingival sequelae [22]. Moran et al. reported one case of partial anterior maxillary necrosis with associated loss of tooth vitality after a 10-mm conventional advancement [21]. Moran et al. also reported one separate case of localized gingival necrosis with bony dehiscence [21]. 

### Aggregate interpretation of the extracted data

Across the included studies, clinically significant maxillary AVN was most frequently reported in the presence of multiple concurrent surgical and vascular factors rather than a single isolated event [17, 19, 21–23]. These included segmented osteotomy, transverse expansion, large maxillary advancement, palatal soft-tissue injury, cleft-related scarring, and compromised collateral circulation [17, 19, 21–23]. No single factor, including DPA ligation, consistently explained all reported ischemic events across the included studies [18–20, 23]. 

## Discussion

Building on previous reviews of reported avascular necrosis or osteonecrosis after orthognathic surgery, the present systematic review focused on cohort studies and case series addressing maxillary avascular necrosis and related ischemic complications after Le Fort I osteotomy [12, 13]. The available evidence demonstrates that postoperative AVN cannot be adequately explained by any single vascular or surgical factor [17–23]. Although several risk factors have been proposed, none was consistently associated with all reported cases [17–23]. Instead, ischemic complications most often occurred in patients with multiple concurrent sources of vascular compromise [17, 19, 21–23]. These findings support interpreting postoperative AVN as a multifactorial complication resulting from cumulative impairment of tissue perfusion rather than isolated injury to a single vessel [6, 7, 11]. 

–One of the most important observations of this review was the inconsistency of the reported risk factors [17–23]. Segmented Le Fort I osteotomy was the most frequently described surgical characteristic among affected patients [17, 19, 22, 23]. However, segmentation alone is unlikely to be sufficient to produce AVN [2, 24]. Multisegment Le Fort I osteotomy is routinely performed without vascular complications in most patients [2]. Intraoperative perfusion studies also show that Le Fort I osteotomy can reduce gingival or bone blood flow without necessarily causing tissue necrosis [24–26]. Therefore, segmentation is better interpreted as one contributor to vascular burden rather than an independent cause of AVN [24, 25]. Similarly, transverse expansion, large maxillary advancement, and previous palatal surgery have each been implicated in individual reports [17, 19, 21, 22]. However, none was universally present among all cases [17–23]. Conversely, clinically significant ischemic complications occasionally developed in the absence of traditionally recognized risk factors [18, 19]. These findings indicate that individual surgical variables have limited explanatory value when considered in isolation [17–23]. 

The relationship between descending palatine artery (DPA) injury and postoperative ischemia further illustrates this concept [18–20, 23]. Preservation of the DPA has traditionally been regarded as an important principle during Le Fort I osteotomy because of its contribution to palatal blood supply [6, 27, 28]. Nevertheless, the findings of this review do not support a direct relationship between DPA sacrifice and AVN [18–20, 23]. DPA ligation was not consistently followed by ischemic complications [18, 20]. Conversely, AVN occasionally occurred despite no recognized palatal vessel injury [19]. Experimental studies have shown that transection of the descending palatine vessels does not inevitably compromise maxillary viability when other soft-tissue pedicles remain intact [9]. Clinical perfusion studies have also shown that DPA ligation does not necessarily reduce gingival blood flow to a critical level [10]. The apparent inconsistency is therefore not contradictory. Rather, it suggests that the effect of DPA injury depends on the integrity of the remaining collateral circulation [6, 7, 11]. DPA injury should therefore be regarded as one component of a broader process of vascular compromise rather than the sole determinant of postoperative AVN [18–20, 23]. 

The findings of this review are more consistent with cumulative vascular compromise than with a single-cause hypothesis [17–23]. None of the proposed risk factors was present in every patient [17–23]. No individual factor consistently predicted postoperative ischemia [17–23]. Instead, most affected patients exhibited several concurrent conditions capable of reducing tissue perfusion [17, 19, 21–23]. These included segmented osteotomy, extensive maxillary movement, palatal soft-tissue injury, cleft-related scarring, and anatomical vascular variation [11, 17, 19, 21–23]. Cadaveric studies have shown that the Le Fort I segment is supplied by several collateral pathways [7, 8]. Recent anatomical work has demonstrated substantial variation in this vascular anatomy [11]. These findings suggest that vascular reserve is not identical among patients [11]. Each factor alone may be well tolerated [9, 10, 24]. However, each may also reduce the functional reserve of the collateral circulation [6, 11, 24, 25]. When several insults coexist, their combined effects may reduce collateral perfusion below the level required for normal healing [6, 11, 17, 19, 21–23]. This interpretation explains both the rarity of AVN and the marked variability in its clinical presentation [12, 13]. 

Another important finding is that postoperative ischemic complications appear to exist along a biological continuum rather than as distinct pathological entities [12, 13]. Mild ischemia may present as tooth discoloration or loss of pulp vitality [20, 29, 30]. Laser Doppler studies have shown that pulpal and gingival blood flow can change after Le Fort I osteotomy, and these changes may persist during the early healing period [29–32]. More severe vascular compromise may result in gingival necrosis or localized bone exposure [17, 19, 21–23]. Persistent hypoperfusion can ultimately progress to dentoalveolar sequestration, partial maxillary necrosis, or complete AVN [17–19, 21–23]. The clinical presentation therefore appears to depend on the severity and duration of postoperative ischemia rather than on different pathogenic mechanisms [12, 13]. This interpretation explains why similar surgical procedures may produce different clinical outcomes among individual patients [11–13]. 

These findings also have practical implications for surgical planning [6, 27, 28, 33]. Prevention should focus on preserving overall tissue perfusion rather than protecting any single vascular structure [6, 7, 9–11]. Every maneuver capable of reducing blood supply may contribute to the overall ischemic burden [6, 24, 25]. Surgeons should therefore minimize unnecessary periosteal stripping, avoid excessive tension on the palatal soft tissues, preserve vascularized soft-tissue attachments whenever possible, and maintain adequate systemic perfusion throughout the procedure [6, 27, 28, 33]. Particular attention should be given to patients with previous palatal surgery, cleft-related scarring, or other conditions that may reduce collateral vascular reserve [21, 22, 34]. 

Several limitations should be acknowledged. First, all included studies were observational, and most were retrospective. Second, the rarity of clinically significant AVN limited both the number and quality of available studies. Third, substantial heterogeneity in surgical techniques, definitions of ischemic complications, and outcome reporting precluded quantitative meta-analysis. In addition, detailed information regarding intraoperative vascular injury, operative variables, and perioperative hemodynamic status was frequently unavailable. Finally, exclusion of isolated case reports improved methodological consistency but may have omitted uncommon clinical presentations. Therefore, the proposed interpretation should be regarded as a biologically plausible conceptual framework derived from the available evidence rather than a definitively proven mechanism.

## Conclusions

Clinically significant maxillary AVN following Le Fort I osteotomy is an exceptionally rare but potentially devastating complication [5, 12, 13]. Segmented osteotomy, extensive maxillary movement, palatal soft-tissue compromise, and previous palatal surgery were the factors most frequently associated with postoperative ischemic complications [17, 19, 21–23]. However, no single factor consistently explained all reported cases, and the role of descending palatine artery injury remains controversial [18–20, 23]. Collectively, the available evidence suggests that postoperative AVN is more likely to result from the combined effects of multiple vascular insults than from isolated injury to a single vessel [6, 7, 9–11]. Further prospective studies are required to clarify the relative contribution of individual risk factors.

## Supplementary Information

Supplementary Material 1: Table S1. JBI Critical Appraisal Checklist for Case Series and study-level assessments. Table S2. JBI Critical Appraisal Checklist for Cohort Studies and study-level assessments. Table S3. Patient-level and aggregate reports of maxillary ischemic complications following maxillary osteotomy.

## Data Availability

No datasets were generated or analysed during the current study.
