# Supplementary material for: Maxillary avascular necrosis after Le Fort I osteotomy: a systematic review of ischemic complications
Source: Maxillofac Plast Reconstr Surg. 2026 Aug 19;48(1):23. doi: 10.1186/s40902-026-00523-x (PMC13493723; doi:10.1186/s40902-026-00523-x)
Supplement: Supplementary file 1 [file 40902_2026_523_MOESM1_ESM.docx]

**JBI critical appraisal: supplementary tables**

The tables present item-level judgments and overall inclusion decisions; no aggregate numerical quality score was calculated.

**Supplementary Table S1. JBI Critical Appraisal Checklist for Case Series and study-level assessments**

| **Item** | **Appraisal criterion** | **Rating** | **Study-level rationale** |
| --- | --- | --- | --- |
| **Q1** | Clear inclusion criteria | **U** | Cases were obtained through a questionnaire/reporting process; prespecified eligibility criteria for an AVN case were not clearly reported. |
| **Q2** | Condition measured consistently and reliably | **U** | Clinical sequelae were described, but a uniform assessment protocol across all reporting surgeons or centres was not demonstrated. |
| **Q3** | Valid identification of the condition | **U** | The cases were clinically compatible with maxillary ischemia/aseptic necrosis, but standardized or validated diagnostic criteria were not specified for all cases. |
| **Q4** | Consecutive inclusion of participants | **N** | Voluntary questionnaire-derived cases do not establish consecutive case ascertainment. |
| **Q5** | Complete inclusion of participants | **N** | Completeness could not be ensured because ascertainment depended on surgeon response and reporting. |
| **Q6** | Clear participant demographics | **Y** | Relevant demographic and case characteristics were reported across the assembled series. |
| **Q7** | Clear clinical information | **Y** | Surgical procedures, ischemic manifestations, and suspected contributing factors were described. |
| **Q8** | Outcomes or follow-up clearly reported | **Y** | Clinical sequelae and management outcomes were reported, although follow-up detail varied among cases. |
| **Q9** | Clear site/clinic demographic information | **U** | The survey context was described, but centre-level characteristics and the source population were not sufficiently detailed for all cases. |
| **Q10** | Appropriate statistical analysis | **Y** | Descriptive synthesis was appropriate for a questionnaire-based series of rare adverse events; no causal effect estimate was attempted. |

Study assessed: Lanigan et al. (1990). Overall appraisal: Include. Y = yes; N = no; U = unclear; NA = not applicable. Item labels are condensed from the JBI checklist for presentation.

*Source: Munn Z, Barker TH, Moola S, et al. Methodological quality of case series studies: an introduction to the JBI critical appraisal tool. JBI Evid Synth. 2020;18:2127–2133.*

**Supplementary Table S2. JBI Critical Appraisal Checklist for Cohort Studies and study-level assessments**

The cohort checklist was applied to five retrospective cohort studies and one prospective cohort study. Items not relevant to an uncontrolled descriptive cohort were marked NA rather than forced into a binary judgment.

| **Item** | **Condensed JBI appraisal criterion** | **Item** | **Condensed JBI appraisal criterion** |
| --- | --- | --- | --- |
| **Q1** | Comparable groups recruited from the same population | **Q7** | Outcome measured validly and reliably |
| **Q2** | Exposure measured similarly in exposed and unexposed groups | **Q8** | Follow-up reported and long enough for the outcome |
| **Q3** | Exposure measured validly and reliably | **Q9** | Follow-up complete or losses described and explored |
| **Q4** | Potential confounding factors identified | **Q10** | Strategies used to address incomplete follow-up |
| **Q5** | Strategies stated to address confounding | **Q11** | Appropriate statistical analysis |
| **Q6** | Participants free of the outcome at baseline/exposure |  |  |

[Study-level appraisal matrix]

| **Study** | **Q1** | **Q2** | **Q3** | **Q4** | **Q5** | **Q6** | **Q7** | **Q8** | **Q9** | **Q10** | **Q11** | **Overall** |
| --- | --- | --- | --- | --- | --- | --- | --- | --- | --- | --- | --- | --- |
| **de Mol van Otterloo et al. (1991)** | NA | NA | Y | NA | NA | Y | Y | Y | U | NA | Y | **Include** |
| **Kramer et al. (2004)** | Y | Y | Y | Y | N | Y | Y | Y | U | NA | U | **Include** |
| **Lee et al. (2016)** | Y | Y | Y | Y | Y | Y | U | Y | N | N | Y | **Include** |
| **Moran et al. (2018)** | Y | Y | Y | N | N | Y | Y | Y | N | N | Y | **Include** |
| **Heggie et al. (2021)** | NA | NA | Y | Y | N | Y | Y | Y | U | NA | Y | **Include** |
| **Tai et al. (2022)** | Y | Y | Y | Y | U | Y | Y | Y | U | NA | Y | **Include** |

Y = yes; N = no; U = unclear; NA = not applicable.

[Key study-level appraisal observations]

| **Study** | **Design** | **Key appraisal observations** |
| --- | --- | --- |
| **de Mol van Otterloo et al. (1991)** | Retrospective descriptive cohort of 410 procedures. | Clinical and operative records supported complication ascertainment, but the report did not form explicit exposure comparison groups and did not fully describe follow-up completeness. |
| **Kramer et al. (2004)** | Prospective cohort of 1,000 consecutive procedures. | Prospective and consecutive inclusion strengthened ascertainment. Risk factors were identified, but adjustment for confounding and handling of incomplete follow-up were not clearly reported. |
| **Lee et al. (2016)** | Retrospective cohort of 1,455 procedures. | Exposure variables were consistently extracted and statistical testing was reported. Tooth discoloration was identified through clinical treatment records rather than a uniform prospective pulp-vitality protocol; follow-up losses were not addressed. |
| **Moran et al. (2018)** | Retrospective cohort of 79 cleft patients. | Eligibility and scheduled postoperative assessment were described. Restriction to complete records and limited control of confounding introduced potential selection and confounding bias. |
| **Heggie et al. (2021)** | Retrospective cohort of 207 cleft patients with detailed review of five events. | All institutional cases were screened and affected patients had at least 3 years of follow-up. The analysis was primarily descriptive and did not adjust for competing vascular or surgical risk factors. |
| **Tai et al. (2022)** | Retrospective cohort of 120 consecutive patients. | Consecutive inclusion, standardized radiographic time points, and 2-year follow-up were strengths. Risk estimates were reported, although the extent of multivariable confounder control and follow-up completeness was not fully clear. |

*Source: JBI Critical Appraisal Checklist for Cohort Studies (2017); Moola S, Munn Z, Tufanaru C, et al. Chapter 7: Systematic reviews of etiology and risk. In: Aromataris E, Munn Z, editors. JBI Manual for Evidence Synthesis. JBI; 2020.*

**Supplementary Table S3. Patient-level and aggregate reports of maxillary ischemic complications following maxillary osteotomy**

Patient-level data were extracted when individual case descriptions were available. Aggregate rows are presented when only cohort-level data were reported; therefore, one row does not necessarily represent one patient.

| **Source article** | **Case** | **Patient / background** | **Procedure / maxillary movement** | **DPA / palatal vascular status** | **Time to ischemia** | **Clinical presentation** | **Management** | **Outcome** |
| --- | --- | --- | --- | --- | --- | --- | --- | --- |
| Lanigan et al. (1990) | 1 | 18F; apertognathia | Le Fort I, 2-piece; palatal splint | NR | 10 weeks | Right maxillary central incisor darkened; non-vital | Endodontic treatment and apicoectomy | NR |
| Lanigan et al. (1990) | 2 | 16M; transverse maxillary deficiency | Le Fort I, 3-piece | DPAs intact | 12 days | Right maxillary central incisor darkened progressively | Endodontic treatment | NR |
| Lanigan et al. (1990) | 3 | 50F; apertognathia | Anterior (Wunderer) + posterior maxillary osteotomies; palatal splint | Left DPA transected | 9 weeks | Papilla slough between left maxillary second premolar and first molar; small bony necrosis | Conservative debridement; approx. 5 x 5 mm bone removed until bleeding | NR |
| Lanigan et al. (1990) | 4 | 25M; vertical maxillary excess + transverse constriction | Le Fort I, 3-piece; intrusion + transverse expansion | DPAs intact | 5-7 days | Necrosis of papillae between lateral incisors and canines bilaterally; canine periodontal defects | NR | Teeth remained vital; residual periodontal defects |
| Lanigan et al. (1990) | 5 | 17M; midfacial retrusion + apertognathia | Le Fort I, 2-piece; 14-mm advancement + posterior intrusion + transverse constriction | Left DPA divided | 6 weeks | Both maxillary central incisors darkened | Endodontic treatment | Teeth treated successfully |
| Lanigan et al. (1990) | 6 | 24F; vertical maxillary excess + apertognathia | Le Fort I, 5-piece; 7-mm intrusion; first premolars extracted | DPAs intact | 2 weeks | Necrosis of marginal gingiva/papillae between central and lateral incisors; alveolar sequestration; central incisors slightly darkened | NR | Central incisors had not required endodontic therapy at report |
| Lanigan et al. (1990) | 7 | 41F; transverse maxillary constriction | Le Fort I, 3-piece; transverse expansion | DPAs intact | 7 days | Sloughing of gingival papillae between lateral incisors and canines bilaterally; periodontal defects | NR | Teeth remained vital; defects stabilized |
| Lanigan et al. (1990) | 8 | 19M; vertical maxillary excess + mandibular retrognathia | Le Fort I, 4-piece; intrusion; BSSO + advancement genioplasty | Clip on left DPA; palatal mucosa intact | 1 week | Gingival recession around central incisors progressing to gingival/alveolar bone loss; central incisors non-vital | Endodontic treatment | Central incisors treated; residual bone loss |
| Lanigan et al. (1990) | 9 | 32F; vertical maxillary excess + mandibular retrognathia | Le Fort I, 3-piece; intrusion; BSSO advancement | NR | 12 weeks | Pain in right maxillary second premolar; non-vital | Attempted endodontic treatment; extraction | Tooth extracted |
| Lanigan et al. (1990) | 10 | 32M; maxillary transverse constriction + mandibular retrognathia | Le Fort I, 4-piece; expansion; extensive bony interference reduction, especially palate | NR | During MMF period | Necrosis of gingiva over right maxillary first premolar; buccal plate involvement | Removal of tooth and buccal plate | Right first premolar and some buccal plate lost |
| Lanigan et al. (1990) | 11 | 18M; vertical maxillary excess, apertognathia, mandibular prognathism | Le Fort I; 14-mm intrusion + 12-mm advancement; mandibular setback | NR | Postoperative day 1 | Blanching/sloughing of maxillary gingiva | Hyperbaric oxygen therapy | Canine gingival recession; loss of left first premolar; endodontic treatment of left first molar |
| Lanigan et al. (1990) | 12 | 30F; transverse constriction + mandibular prognathism/laterognathia; heavy smoker | Le Fort I, 2-piece; transverse expansion; BSSO setback/rotation; midpalatal split; electrocautery incision | DPAs appeared intact; anterior palatal perforation | 1 week | Anterior labial gingival slough with exposed labial bone | Hyperbaric oxygen attempted but stopped after two sessions | 4-5 mm alveolar crest loss; central incisors extracted |
| Lanigan et al. (1990) | 13 | 21F; apertognathia | Le Fort I, 4-piece; intrusion; palatal splint | NR | 1 week | Recession around left maxillary anterior teeth | NR | Loss of left lateral incisor, canine, and supporting alveolar bone |
| Lanigan et al. (1990) | 14 | 28M; vertical maxillary excess | Le Fort I, 3-piece; intrusion | DPAs intact; no palatal perforation | 2-3 weeks | Sloughing between canines and first premolars bilaterally; alveolar bone loss around canines | Extraction of both maxillary canines at 3 years; fixed bridge placement | Maxillary canines extracted at 3 years; fixed bridge |
| Lanigan et al. (1990) | 15 | 35M; apertognathia | Le Fort I, 3-piece; palatal splint | DPAs severed | 4 weeks | Purulent drainage; exposed palatal bony segment containing left premolars; mobile/free-floating molar segment | Palatal splint removal | Palatal segment sloughed; large oroantral fistula |
| Lanigan et al. (1990) | 16 | 27F; vertical maxillary excess, retrusion, mandibular retrognathia | Le Fort I, 3-piece; intrusion, advancement, expansion; mandibular advancement + genioplasty | Anterior palatal mucosal tear; mucosa immediately white/ischemic | Intraoperative to first 2 weeks | Anterior buccal/palatal ischemia; marginal gingival necrosis; anterior teeth non-vital | NR | Progressive recession; 3-4 mm root exposure over five anterior teeth; stable at report |
| Lanigan et al. (1990) | 17 | 36F; apertognathia + mandibular retrognathia; sinus history; chronic periodontal disease | Le Fort I, 3-piece; intrusion + transverse expansion; BSSO advancement; reoperation for hemorrhage | Both DPAs cut/clipped; no palatal tears; severe hemorrhage and redownfracture | 48 hours | Anterior gingival sloughing; periodontal loss between left canine and first premolar | Hemorrhage control; free gingival grafting | Loss of left canine, first premolar and alveolar bone; oroantral fistula closed spontaneously |
| Lanigan et al. (1990) | 18 | 48F; posterior maxillary hyperplasia | Le Fort I, 3-piece; posterior intrusion; relieved palatal strap | DPAs intact | 1 week | Right vestibular dehiscence; exposed alveolar bone became necrotic | Removal of mobile teeth/bone; buccal flap closure | Loss of right canine and first/second premolars; oroantral fistula closed surgically |
| Lanigan et al. (1990) | 19 | 25F; vertical maxillary excess + mandibular prognathism | Le Fort I with intrusion + mandibular setback; horseshoe palatal cut due to deep vault | Small 1-cm anterior left palatal mucosal tear | 2 days | Necrosis of gingiva and alveolar bone in left anterior maxilla | NR | Loss of left canine, central and lateral incisors, and supporting bone |
| Lanigan et al. (1990) | 20 | 32F; vertical maxillary excess | Le Fort I, 4-piece; intrusion and retrusion of anterior segment | No palatal perforation | 2 weeks (patient noticed dark gingiva at 1 week) | Right anterior maxillary necrosis | Hyperbaric oxygen therapy, 24 sessions | Loss of right central/lateral incisors, canine, and surrounding buccal alveolar bone |
| Lanigan et al. (1990) | 21 | 26F; vertical maxillary excess | Le Fort I, 3-piece; intrusion and setback of anterior segment to close edentulous space | DPAs intact; no palatal perforation | 8 days | Anterior gingival necrosis; labial bone around three anterior teeth necrotic | Debridement at 2 weeks; anterior teeth removed | Half of labial alveolar bone lost; palatal bone and soft tissue pedicle retained |
| Lanigan et al. (1990) | 22 | 32F; vertical maxillary excess, apertognathia, mandibular retrognathia | Le Fort I, 2-piece; maxillary first premolars removed; superior and posterior repositioning; BSSO advancement + genioplasty | NR | 2 days | Dusty gray labial mucosa/gingiva in left anterior maxilla; exposed alveolar bone | Extraction of the left central and lateral incisors and canine; other management NR | Significant labial bone loss; left central/lateral incisor and canine extracted |
| Lanigan et al. (1990) | 23 | 19F; vertical maxillary excess + apertognathia | Le Fort I, 3-piece; first premolars extracted | Left DPA nicked; clip placed | 2 weeks | Left anterior dehiscence; sloughing papillae from central incisor to canine | NR | Loss of left central/lateral incisors, canine, and associated alveolar bone |
| Lanigan et al. (1990) | 24 | 42M; maxillary transverse constriction | Le Fort I, 4-piece; expansion; palatal splint | DPAs intact; no palatal mucosal perforation | Approx. 7 days | Slough of left anterior gingiva and bone | Hyperbaric oxygen therapy | Loss of left central/lateral incisors, canine, and surrounding alveolar bone |
| Lanigan et al. (1990) | 25 | 47F; vertical maxillary excess + mandibular retrognathia | Le Fort I, 2-piece; intrusion + transverse expansion; skeletal suspension wires | Left DPA bleeding clipped/cauterized; tight right piriform wire compressed soft tissue | 2 days noted compression; later AVN | Right anterior maxillary AVN | Removal of the tight right piriform fossa wire | Loss of central/lateral incisors, canine, and supporting bone |
| Lanigan et al. (1990) | 26 | 39F; vertical maxillary excess | Le Fort I, 4-piece; intrusion + transverse expansion | Both DPAs cut | 7 days | Necrosis of periodontal tissues between central incisors and left premolars | Penicillin 2 weeks | By 3 months, central incisors, left premolars, and supporting bone lost |
| Lanigan et al. (1990) | 27 | 33M; vertical maxillary excess + transverse constriction | Le Fort I, 2-piece; 3-mm intrusion, 2-mm retrusion, 12-mm posterior expansion | DPAs transected | First 3 days | Anterior attached gingiva changed tan and became necrotic | Extraction of mobile incisors | Loss of right central/lateral incisors and left central incisor; left lateral already absent |
| Lanigan et al. (1990) | 28 | 16F; bilateral cleft lip/palate; heavily scarred palate; 17 prior operations | Right hemi-Le Fort I + modified left hemi-Le Fort III; bone grafting; BSSO; genioplasty; turbinectomies | Limited incisions/labial pedicle; DPA NR | 12 days | Necrosis of palatal flap to premaxillary segment; marginal denudation; premaxilla viable | NR | Maxillary relapse, residual fistulae, loss of cleft bone grafts and canines/first premolars |
| Lanigan et al. (1990) | 29 | 22F; vertical maxillary excess + transverse constriction | Le Fort I, 2-piece; 9-mm anterior/6-mm posterior intrusion; 6-mm posterior expansion | DPAs sacrificed; no palatal perforation | Within 10 days | Cyanosis and sloughing of anterior gingiva and bone, especially right | HBOT; tetracycline fluorescence; debridement at 6 weeks; flap closure; later iliac graft and vestibuloplasty | Nonunion noted; central incisors extracted; canine later extracted; later reconstruction |
| Lanigan et al. (1990) | 30 | 19F; unilateral cleft lip/palate; prior alveolar cleft graft; extensive scarring from pushback | Le Fort I, one-piece advancement | Both DPAs absent, likely from previous palatal surgery | Intraoperative to postoperative | Marked cyanosis; sloughing buccal mucosa/gingiva; slow alveolar sequestration | Extraction of the right first premolar and canine and the left central incisor at 18 months | Right first premolar/canine and left central incisor extracted; right central/lateral non-vital with bone loss |
| Lanigan et al. (1990) | 31 | 53F; vertical maxillary excess + mandibular retrognathia | Le Fort I, 4-piece; BSSO advancement | DPAs intact | 10 days | Gingival recession around right anterior segment and left central incisor; later fistula; non-vital segment/root resorption | Extractions and bone debridement at 8 months; fistula closure at 17 months | Multiple anterior teeth extracted; residual tooth root resorption |
| Lanigan et al. (1990) | 32 | 46F; bimaxillary protrusion | Wassmund anterior maxillary osteotomy via vertical buccal incisions/tunneling | Palatal mucosa perforated; greater palatine arteries sectioned with profuse bleeding | 5 days | Ischemia/cyanosis of gingiva and palatal mucosa; progressive slough of anterior maxilla | Debridement | Loss of anterior maxillary teeth, alveolar bone, soft tissue; persistent small anterior oronasal fistula |
| Lanigan et al. (1990) | 33 | 28F; horizontal maxillary excess | Anterior maxillary osteotomy with setback via downfracture; tight full-coverage palatal splint | Anterior segment pedicled to palatal mucosa; DPA NR | Approx. 1.5 weeks | Gingival sloughing; exposed bone; progressive tooth loosening | Antibiotics; attempted mucogingival flap; debridement; fistula closure; later implants | Loss of most anterior maxillary teeth; large oronasoantral fistula closed; implant-supported prosthesis |
| Lanigan et al. (1990) | 34 | 33F; apertognathia + mandibular retrognathia | Le Fort I, 3-piece; 4-mm intrusion; 15-mm posterior expansion; BSSO advancement; prolonged hypotensive anesthesia | Left DPA appeared stretched but intact; no palatal perforation | Immediate to day 4 | Blue discoloration progressing to frank maxillary AVN with palatal involvement | Redownfracture; rigid fixation; HBOT 20 sessions; later iliac bone graft, palatal/lip flaps, implants, tongue flap | Extensive tooth/alveolar/palatal bone loss; reconstruction partly successful; residual fistula |
| Lanigan et al. (1990) | 35 | 13M; Crouzon disease; previous Le Fort III advancement | Le Fort I advancement | DPAs severed; adherent soft tissue detached during traction | Intraoperative onward | Maxillary mucosa became white/no bleeding; anterior teeth, gingiva and bone sloughed | Long-term follow-up; specific treatment NR | Loss from right first premolar to left first premolar; continuing periodontal loss/root resorption suspected |
| Lanigan et al. (1990) | 36 | 32M; facial asymmetry from left maxillary/mandibular vertical alveolar hyperplasia | Planned left hemi-Le Fort I intrusion/constriction + mandibular osteotomies; palatal incision; pressure packing; later embolization | Left DPA sectioned/cauterized; posterior buccal pedicle tore; external carotid/maxillary embolization | 2 days after embolization | Ischemia of left maxillary segment; later exfoliation of teeth and buccal bone | HBOT 20 sessions; hemorrhage control/embolization | Loss of left maxillary teeth central incisor-second molar and buccal bone; 50-75% segment preserved; small oronasal communication |
| de Mol van Otterloo et al. (1991) | 1 | NR; non-cleft Le Fort I cohort (410 procedures) | Three-piece Le Fort I osteotomy | Individual vascular status NR; damaged greater palatine arteries were ligated in six haemorrhagic cases without further complications, and overlap with the necrosis case was not reported. | NR | Devitalization of a cuspid and buccal cortical bone necrosis | NR | NR |
| Kramer et al. (2004) | AVN-1 | NR; prospective Le Fort I cohort (1,000 patients) | Le Fort I with transversal segmentation; 9-mm anterior mobilization | Individual palatal vessel status NR; the authors reported that some ischemic complications occurred without recognized intraoperative palatal vessel injury. | NR | Partial maxillary/alveolar necrosis | NR | NR |
| Kramer et al. (2004) | AVN-2 | NR; prospective Le Fort I cohort (1,000 patients) | Le Fort I; 10-mm anterior displacement | Individual palatal vessel status NR; the authors reported that some ischemic complications occurred without recognized intraoperative palatal vessel injury. | NR | Subtotal aseptic necrosis of maxillary alveolar process | NR | Clinical appearance at 3 months was illustrated; longer-term outcome NR. |
| Kramer et al. (2004) | Gingival retraction cases | 8 patients; cohort-level data | Le Fort I osteotomy | NR | Early postoperative | Gingival retraction without osseous AVN | NR | NR |
| Lee et al. (2016) | 39 patients / 48 maxillary teeth | Orthognathic surgery cohort; 1339 double-jaw and 116 single-jaw surgeries | Orthognathic surgery involving Le Fort I; maxillary tooth discolouration occurred in 39 patients (48 teeth). | DPA ligation was associated with maxillary tooth discolouration: 24/567 patients with ligation versus 15/772 without ligation. | Average recognition: 6 months | Maxillary tooth discolouration with necrotic pulp; bone or soft-tissue AVN was not evaluated as an outcome. | Root canal treatment and tooth bleaching | Long-term outcome NR |
| Moran et al. (2018) | 1 | Bilateral cleft lip/palate | Conventional Le Fort I advancement, 10 mm | DPA not evaluated | Postoperative period | Partial anterior maxillary necrosis; five anterior teeth non-vital/mobile | Surgical debridement; iliac crest bone graft; implant placement | Reconstructed/recovered |
| Moran et al. (2018) | 2 | Bilateral cleft lip/palate | Conventional Le Fort I advancement, 9 mm | DPA not evaluated | Postoperative period | Localized gingival necrosis around UL1 with bony dehiscence | Oral hygiene instruction; surgical debridement; soft-tissue graft | Long-term outcome NR after debridement and soft-tissue grafting. |
| Heggie et al. (2021) | 1 | 22M; right CLP; palatal transpositional flap at 13 y for residual midline fistula | Le Fort I advancement/impaction with segmentalization between right central incisor and canine for expansion | DPA not specified; previous palatal flap | 9 days | Labial exposed bone at cleft site, progressed canine-to-canine | Removal of central incisors and sequestrating labial plate at 4 months; removal of canines at 17 months; later onlay grafting and dental implants. | Tooth loss and later implant rehabilitation |
| Heggie et al. (2021) | 2 | 18M; right CLP; palatal transpositional flap at 13 y for oronasal fistula | Le Fort I advancement/impaction with segmentalization between central incisors for expansion | DPA not specified; previous palatal flap | Postoperative day 1 | Ischemic region from right canine to left central incisor | HBOT for 1 week | Gingiva/papillae regenerated with recession; central incisors removed 12 years later due to internal resorption/ankylosis; implants |
| Heggie et al. (2021) | 3 | 23M; left CLP; pharyngeal flap at 8 y for velopharyngeal insufficiency | Single-segment Le Fort I advancement | DPA not specified; previous pharyngeal flap | Postoperative day 1; exposed bone at 2-3 weeks | Anterior segment soft-tissue discoloration; exposed bone along left canine/premolar gingival margin | HBOT from postoperative day 2 for 2 weeks | Moderate gingival recession; dentition stable at 3 years |
| Heggie et al. (2021) | 4 | 18M; bilateral CLP; alveolar bone graft with transpositional palatal flap elevation at 9 y to close large fistula | Single-segment Le Fort I advancement with partial release of pharyngeal flap | DPA not specified; previous palatal flap surgery | Postoperative day 1 | Marked ischemia/purple discoloration of anterior maxillary gingiva | HBOT for 3 days | Soft-tissue recovery; mucosa regenerated; teeth retained |
| Heggie et al. (2021) | 5 | 21M; bilateral CLP; palatal fistula closure with transpositional palatal flap at 19 y | Single-segment Le Fort I advancement | DPA not specified; previous palatal flap surgery | Immediate postoperative | Marked soft-tissue necrosis of anterior gingiva from left canine to right central incisor | Incisor splinting; HBOT unavailable; left central incisor/canine removal and minor debridement at 4 weeks; implants | Left central incisor and canine lost; right central incisor retained with recession |
| Tai et al. (2022) | 4 AVN cases (aggregate) | 120-patient cohort undergoing segmented Le Fort I osteotomy for bimaxillary dentoalveolar protrusion | 3- or 4-piece Le Fort I with anterior segment repositioning | DPA transection reported in 6/120 overall; individual overlap with AVN not reported | Classified as an early complication; individual onset NR | Anterior maxillary segment AVN ranging from periodontal necrosis to dentoalveolar sequestration and loss of two incisors | NR | All four patients with AVN developed gingival recession; other individual outcomes NR. |

Abbreviations: AVN, avascular necrosis; BSSO, bilateral sagittal split osteotomy; CLP, cleft lip and palate; DPA, descending palatine artery; HBOT, hyperbaric oxygen therapy; MMF, maxillomandibular fixation; NR, not reported; UL1, upper left central incisor.

Note: Lee et al. evaluated maxillary tooth discolouration/pulp necrosis rather than clinically evident maxillary bone or soft-tissue AVN. Tai et al. reported four AVN events but did not provide individual patient-level details or overlap between DPA transection and AVN. Lanigan et al. presented 36 best-documented cases selected from 51 events reported through a questionnaire survey; consecutive and complete case ascertainment could not be verified.
